# Alglucosidase alfa: 5 years of experience in late-onset Pompe disease

**DOI:** 10.1186/1471-2474-14-S2-O9

**Published:** 2013-05-29

**Authors:** Benedikt Schoser

**Affiliations:** 1 Friedrich-Baur Institute, Department of Neurology, University of Munich, Munich, Germany

## 

Glycogen storage disease type 2, Pompe disease, is a progressive muscle disorder with a wide range of phenotypic presentations, caused by an inherited deficiency of the enzyme acid alpha-glucosidase. Although only a few patients have been treated with recombinant human alpha-glucosidase from rabbit milk since 2004, enzyme replacement therapy (ERT) with alglucosidase alfa has been licensed for the treatment of Pompe disease since 2006. Here, a systematic review [1] evaluates the clinical efficacy and safety of alglucosidase alfa treatment in juvenile and adult patients with late-onset Pompe disease (LOPD). Studies of alglucosidase alfa treatment in patients with LOPD, published up to October 2012, were identified using an electronic search of the EMBASE and MEDLINE databases, and manual searches of the reference lists. Data on ERT outcomes were extracted from the selected papers and analyzed descriptively. No statistical analyses were performed owing to data heterogeneity. Twenty-two studies containing clinical data from 437 LOPD patients were analyzed. Overall, at least two-thirds of patients were stabilized or exhibited improvements in creatine kinase levels, and muscular and/or respiratory function following treatment with alglucosidase alfa. Enzyme replacement therapy was well tolerated; the majority of adverse events were mild or moderate infusion-related reactions. Alglucosidase alfa treatment offers an effective and well tolerated treatment that attenuates the progression of LOPD in the majority of patients. Although first insights are upcoming, further research is required to investigate reliable prognostic factors such as age at treatment start, phenotypic presentation, and genotypic characteristics, of which may enable better clinical and therapeutic management of LOPD patients.
